# Gum Rosin in Medical and Pharmaceutical Applications: From Conventional Uses to Modern Advancements

**DOI:** 10.3390/ma18102266

**Published:** 2025-05-13

**Authors:** Sonita Afrita Purba Siboro, Sabrina Aufar Salma, Syuhada, Kurnia Sari Setio Putri, Frita Yuliati, Won-Ki Lee, Kwon-Taek Lim

**Affiliations:** 1 Polymer Technology, National Research and Innovation Agency, Tangerang 15346, Indonesia; soni009@brin.go.id (S.A.P.S.); sabr003@brin.go.id (S.A.S.); syuh001@brin.go.id (S.);; 2 Faculty of Pharmacy, Universitas Indonesia, Depok 16424, Indonesia; kurnia.putri@farmasi.ui.ac.id; 3 Department of Polymer Engineering, Pukyong National University, Busan 48513, Republic of Korea; wonki@pknu.ac.kr; 4 Institute of Display Semiconductor Technology, Pukyong National University, Busan 48513, Republic of Korea

**Keywords:** rosin, pharmaceutical excipient, controlled drug release, targeted drug delivery, nanocarriers

## Abstract

Gum rosin and its derivatives have been used traditionally in coatings and adhesives and are now increasingly applied in diverse medical and pharmaceutical fields. Owing to its film-forming ability, hydrophobic nature, biocompatibility, and ease of chemical modification, gum rosin has emerged as a promising excipient for controlled drug release, targeted drug delivery, and other biomedical applications. This review summarizes the evolution of gum rosin applications, from its conventional roles to its modern utilization in nanocarriers, transdermal systems, and other advanced drug delivery platforms. In addition, we discuss the challenges related to allergenicity, brittleness, and excessive hydrophobicity and propose strategies (such as chemical modification and polymer blending) to overcome these issues. This review provides a reference framework for researchers developing new rosin-based materials in pharmaceutical sciences.

## 1. Introduction

Gum rosin, also known as rosin or colophony, is a natural, renewable resin obtained from the distillation of pine sap. Chemically, gum rosin consists primarily of resin acids (about 90–95%), predominantly abietic acid and its isomers, along with neutral compounds (resenes) [1,2]. These resin acids are solid and brittle yet possess desirable properties such as film-forming ability, biocompatibility, and biodegradability [3]. Meanwhile, the molecular structure of rosin acids (abietane-type diterpenes) provides sites for chemical modifications, which has enabled the development of numerous rosin derivatives tailored for specific applications [4]. Historically, gum rosin found use in a variety of industries due to its adhesive and film-forming characteristics, for example in varnishes, coatings, and adhesives [5,6]. In medical and pharmaceutical contexts, its conventional uses were relatively limited but significant. For example, rosin has long been used as a tackifier in adhesives for surgical tapes and plasters [7]. Its stickiness helps medical dressings adhere to skin, although this traditional use is sometimes accompanied by skin sensitivity issues. Rosin has also been an ingredient in dental formulations (such as cavity varnishes) and in certain topical preparations. In addition, food-grade hydrogenated rosin esters have been used as components of chewing gum bases [8], and by extension rosin has indirectly been present in some medicated chewing gums. These conventional applications promote rosin’s ability to form a durable film or sticky matrix, but they did not fully exploit its potential as a polymeric excipient in sophisticated drug delivery systems.

Interest in gum rosin as a pharmaceutical excipient has grown as researchers recognized its unique combination of properties. Rosin is highly hydrophobic, which makes it an attractive candidate for forming moisture-resistant coatings or matrices to sustain drug release [9]. At the same time, it is a naturally derived, biodegradable substance, aligning with the current drive toward more ecofriendly and biocompatible materials in drug formulation [1]. Furthermore, rosin can be readily modified chemically (for example, through hydrogenation, esterification, or polymerization) to improve its performance or reduce undesirable traits. Given these advantages, recent research has expanded rosin’s role from traditional uses to modern pharmaceutical applications. Nowadays, rosin has been used widely in biomedical applications due to its availability, cost-effectiveness, and inherent properties such as biocompatibility, biodegradability, and chemical versatility [3,10,11]. A study by Mardiah et al. (2023) showed that rosin is biodegradable and exhibits good film-forming capabilities, and so can be used in biomedical applications, such as antibacterial coatings [12]. To further enhance the performance, numerous chemical modifications have been introduced. For example, a study synthesized water-soluble rosin-PEG derivatives using PEG 1500, achieving complete water solubility and a melting point of 40.6 °C. These derivatives improved wettability and pH-dependent aqueous solubility, making it suitable for controlled drug release applications [13]. Additionally, rosin’s capacity to be transformed into polymerizable monomers has been applied in advanced DDS, including controlled and targeted drug delivery [14]. These modifications of rosin enhance its functionality as a coating agent in various dosage forms and enable its integration into nanoparticles for controlled drug release [1,15].

The advancements in applications of rosin and the derivatives are already summarized in several review articles [1,10,16]. Mahendra (2019), for example, presented a broad overview of rosin’s properties and uses across multiple industries, emphasizing its role as a precursor in paints, inks, adhesives, and other material applications [16]. Kugler et al. (2019) compiled advances in rosin-based chemicals up to 2018, covering everything from low-molecular-weight derivatives (resins, monomers, curing agents, surfactants, and even some medicinally relevant compounds) to rosin-derived polymers for diverse end uses [10]. In a more pharmacognosy-oriented survey, Li and Chen (2024) emphasized rosin’s traditional medicinal uses and phytochemistry, documenting the long history in folk medicine (e.g., treating wounds, burns, and abscesses) and reviewing its bioactive constituents and pharmacological activities [17]. An earlier pharmaceutical-focused review by Yadav et al. (2016) examined rosin and its derivatives as biomaterials in drug formulations, describing their film-forming, coating, and matrix roles in novel drug delivery systems [1]. While these works provide a foundation, their scopes are either broad (covering general industrial or chemical aspects) or centered on traditional uses and earlier generation drug delivery approaches. In contrast, the present review concentrates specifically on modern pharmaceutical and biomedical applications of gum rosin, filling a gap left by the above literature. Our focus is on recent advancements (particularly post-2019) in rosin-based excipients and biomaterials, including contemporary drug delivery platforms and medical devices that promote rosin’s unique film-forming and biocompatible properties. We incorporate the latest findings to date, thereby extending the discussion beyond the 2008–2018 period covered by Kugler et al. and updating the pharmaceutical context that Yadav et al. explored nearly a decade ago. Importantly, we also address the practical challenges that arise when translating rosin into modern biomedical use (such as its inherent brittleness and allergenic potential), along with strategies to overcome these issues. Approaches like chemical modification, derivatization, and polymer blending are discussed as means to enhance rosin’s stability, reduce allergenicity, and improve its functional performance in drug delivery and tissue-contact applications. By critically evaluating these recent developments, this review offers a timely and focused perspective that complements earlier reviews, aiming to guide researchers in harnessing gum rosin’s full potential in current and future pharmaceutical innovations.

## 2. Chemical and Physical Properties

**Figure 1:**
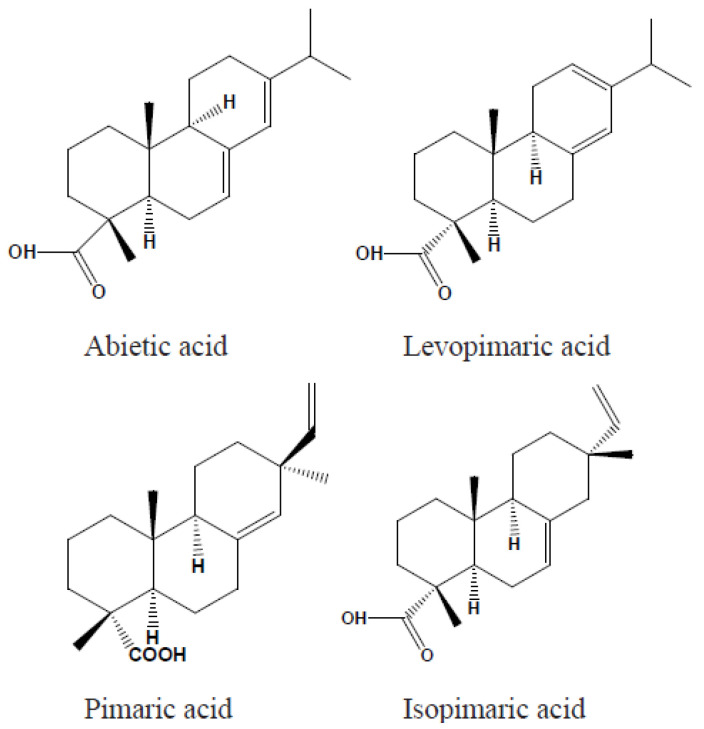
Chemical structures of abietic-type and pimaric-type rosin acids.

Gum rosin is defined as the non-volatile residue obtained from the distillation of pine tree sap. This sap is sourced from various species of pine. One important characteristic of rosin is its low toxicity, which show the suitability for use in food, beverage, and pharmaceutical applications [10,16,18]. Typically, gum rosin comprises 90–95% resin acids, which consist of a mixture of abietic-type and pimaric-type acids, while the remaining fraction is made up of non-acidic substances [1]. Rosin acids are tricyclic diterpene monocarboxylic acids built around a phenanthrene nucleus and are generally represented by the molecular formula C_(20)_H_(30)_O_2_ [10]. This complex structure features two primary reactive sites: the carbon–carbon double bonds and the carboxyl group. In abietic acids, the double bonds are conjugated, which is not the case for pimaric acids. Notable examples of abietic-type acids include abietic acid, neoabietic acid, palustric acid, and levopimaric acid. Moreover, rosin may contain trace amounts of aromatic dehydroabietic acid, and its overall composition can vary with the botanical and geographical origin of the resin. These compositional variations can complicate the precise quantification of individual resin acids. Figure 1 illustrates the chemical structures of representative abietic and pimaric rosin acids [1].

Gum rosin can be classified based on the color and softening point. This color variation depends on factors such as purity, age, and the presence of oxidized products or impurities. Generally, a decrease in the softening point correlates with a darker color. Lighter shades of rosin typically have higher softening points and acid numbers, whereas darker rosin tends to contain increased amounts of unsaponifiable matter, alcohol-insoluble components, and ash. In addition, the color intensity of rosin is related to the abietic acid content. Darker rosin indicates a higher concentration of abietic acid [1,19]. Furthermore, rosin has an amorphous (non-crystalline) nature and is inherently brittle, tending to crack or pulverize under stress. It behaves as a thermoplastic material, softening upon heating and hardening upon cooling. Rosin starts to soften at about 80 °C, whereas crystallized rosin melts in the range of 110–130 °C [20]. Upon cooling, the molten rosin re-solidifies into an amorphous, glass-like solid.

The relative proportions of abietic-type (abietane-structured) acids versus pimaric-type (pimarane-structured) acids in rosin can vary depending on the botanical source. Different pine species yield oleoresins with distinct resin acid profiles. For example, rosin from *Pinus massoniana* (widely tapped in China) contains roughly 87 wt% abietane-type acids and only ~13% pimarane-type acids, whereas *Pinus merkusii* (Indonesia) rosin may have as low as 64% abietane-type and ~36% pimarane-type acids. *Pinus elliottii* (the source of most Brazilian gum rosin) is intermediate, typically ~73% abietane-type versus ~27% pimarane-type resin acids [10]. In practical terms, this means that some species (e.g., *P. massoniana*, *P. halepensis*) produce rosin dominated by abietic-type components, while others (like *P. merkusii*) yield a higher fraction of pimaric-type acids. This compositional variability is inherent to rosin’s natural origin and classifies it as a UVCB (“Unknown or Variable composition, Complex reaction products or Biological material”) substance [20].

## 3. General Uses

**Figure 2:**
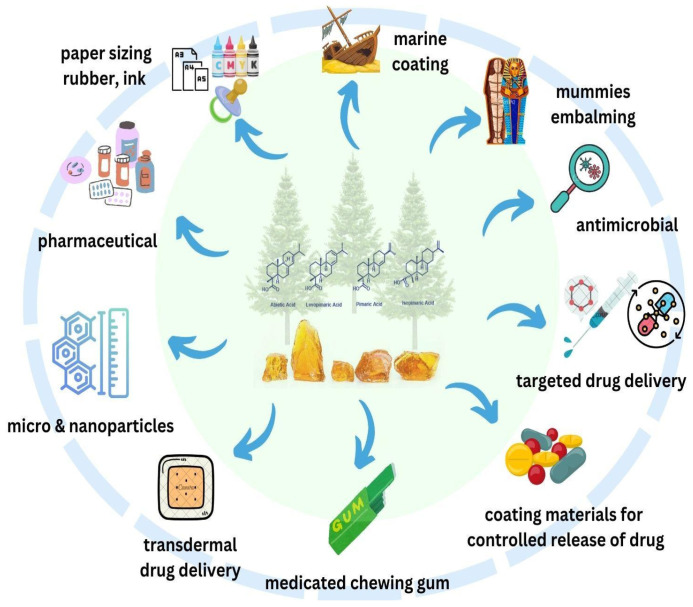
The diverse applications of gum rosin.

Rosin and its derivatives can be employed in both solid and dissolved states, serving many purposes in their applications, as described in Figure 2. The summary of general application can be seen in Table 1. Ongoing researches are still being performed to improve the performances and to find wider applications.

## 4. Medical and Pharmaceutical Applications

It is of interest that rosin and its derivates can also be utilized in medical fields. Researchers have identified various functions of rosin gum in pharmaceutical applications, as detailed below.

### 4.1. Antimicrobial Agents

**Figure 3:**
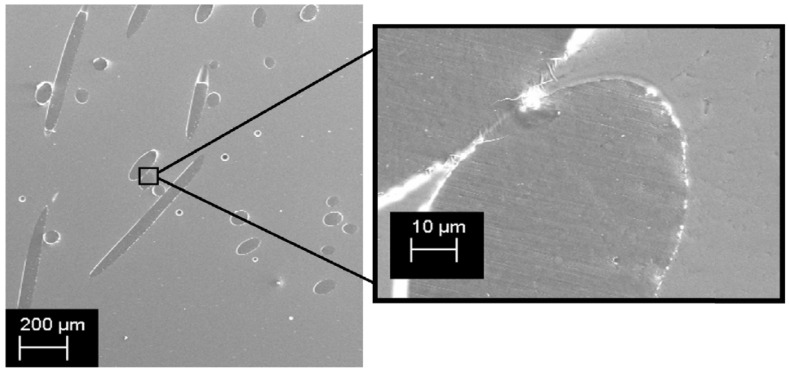
Cross-section SEM images of PE fibers with rosin (fPE10-160). Reproduced from ref. (Kanerva et al., 2019) with permission [43]. Copyright © 2013, Elsevier.

Pure spruce wood, a natural source of rosin, exhibits antibacterial effects against pathogens such as *Escherichia coli*, *Streptococcus pneumoniae*, and *Salmonella enterica* [36]. Gamma irradiation has further been shown to enhance these antimicrobial properties [37]. The antibacterial effect is primarily attributed to the presence of abietic-type resin acids in rosin, which disrupt bacterial cell walls (particularly in *Staphylococcus aureus*), thereby impeding cellular energy generation [38]. Despite these promising properties, the high hydrophobicity of unmodified rosin limits its antimicrobial effectiveness in aqueous environments. To overcome this challenge, rosin has been chemically modified into its maleic anhydride adduct, which improves water solubility and enhances bioinhibitory performance against methicillin-resistant *Staphylococcus aureus* [39]. Moreover, blending rosin with bio-based polymers such as cellulose acetate, poly (butylene succinate), and starch has yielded formulations with significant antimicrobial activity [24,40,41]. It is important to note that the botanical origin of rosin, its incorporation into polymer matrices, and the mode of bacterial exposure can substantially influence its antibacterial response [42]. For example, incorporating 10–20% rosin into polymers such as polyethylene (PE) to produce fibers or filaments has resulted in materials with both robust mechanical properties and effective antimicrobial performance, as evidenced by SEM images in Figure 3 demonstrating good compatibility between rosin and PE and effective inhibition of *E. coli* and *S. aureus* [43].

Additional modification strategies have focused on synthesizing biodegradable cationic antimicrobial compounds by combining rosin acid with quaternary ammonium groups. These cationic groups promote rapid adhesion to negatively charged bacterial cell surfaces, leading to cell lysis, and they preferentially target microbial cells over mammalian cells, thereby reducing the risk of resistance development. One study demonstrated that maleopimaric acid quaternary ammonium cations (MPA-N+) chemically bonded onto cotton textile surfaces (CT-g-MPA-N+) produced a wound dressing with potent antibacterial activity against both Gram-negative bacteria (e.g., *E. coli*, *Pseudomonas aeruginosa*) and Gram-positive bacteria (e.g., *S. aureus*). This modified dressing maintained its antibacterial effectiveness even after prolonged immersion in phosphate-buffered saline and inhibited bacterial biofilm formation [44,45]. Furthermore, Jindal et al. (2017) developed an iron oxide nanogel using a gum rosin–acrylamide copolymer matrix, which demonstrated significant antibacterial efficacy against both *S. aureus* and *E. coli*, with a notably stronger effect against *S. aureus*, a result attributed to differences in cell membrane charge that enhance interactions with the nanogel components [46]. Recent study by Bezzekhami et al. (2023) using nanoarchitectonics of starch nanoparticles rosin showed the enhanced antimicrobial activity against several strains (Gram-positive, Gram-negative, and yeast), with the inhibition zone directly proportional to the degree of substitution, indicating their promising potential in biomedical and food-packaging applications [24].

Preliminary clinical studies have started to validate rosin’s antimicrobial benefits. For example, a randomized trial on chronic pressure ulcers found significantly greater healing rates with a rosin-based resin salve (92% of ulcers completely healed) compared to standard care (44%) [47]. Another study reported a 100% healing rate of chronic surgical wounds using a 10% rosin salve, with wounds healing in an average of 43 days [48]. These outcomes illustrate rosin’s therapeutic potential in infection management, though larger-scale clinical trials are needed to confirm efficacy and safety.

### 4.2. Anticancer Agents

Beside antimicrobial activity, several studies also showed that modification of gum rosin resulted substance with anti-cancer activity. A study performed by Fei et al. (2019) presented the rational synthesis of two chiral copper(II) complexes, [CuL_4_Cl]Cl·2CH_2_Cl_2_·H_2_O (1) and [CuL_4_Br]Br·2CH_2_Cl_2_ (2), derived from rosin and ligand L (2-amino-5-dehydroabietyl-1,3,4-thiadiazole) [49]. The obtained modified rosin exhibited significant in vitro and in vivo anticancer activities with tolerable toxicities. It induced cell death in MCF-7 cells through a combination of G1 phase arrest, apoptosis (involving both extrinsic and intrinsic pathways), anti-metastasis, anti-angiogenesis, and damage to DNA, proteins, and lipids. Autophagy, mediated by oxidative stress, was also evident [49]. Another study explored antioxidant activity of rosin-derived crude methanol extract (RD-CME) using 2,2-Diphenyl-1-picrylhydrazyl (DPPH) assay and assessed cytotoxicity of RD-CME against two types of breast cancer cells (MCF-7 and MDA-MB231) using 3-(4,5-dimethylthiazol-2-yl)-2,5-diphenyltetrazolium bromide (MTT) colorimetric. The results proved that a low concentration of RD-CME showed high antioxidant scavenging activities. Furthermore, the cytotoxicity of test showed that RD-CME exhibited a high cytotoxicity against the two cancer cells, even higher than the control drug (doxorubicin) [50].

To date, there are no reported clinical trials investigating gum rosin-derived compounds as anticancer treatments. Current evidence of anticancer activity arises solely from in vitro studies and animal models. While these preclinical results are promising, showing that rosin abietane diterpenoids can induce tumor cell death via mechanisms like apoptosis and cell-cycle arrest, clinical validation in cancer patients is still lacking. Thus, the therapeutic anticancer potential of rosin-based compounds remains unproven in a clinical setting.

### 4.3. Corrosion Retardant for Biodegradable Implants

The use of biodegradable implants in bone screws, plates, and pins is preferable to avoid the need of removing implants after they are no longer needed. Magnesium is a potential candidate, since it is degradable in physiological environment, and it is naturally found in bone tissues. However, magnesium degrades very quickly in aqueous environment, leading to quick formation of hydrogen gas and diminishing its mechanical integrity. Coating materials made of mixtures of gum rosin and beeswax were found to reduce the corrosion rate of magnesium alloys in NaCl solution. The coating itself is expected to be biodegradable and harmless to human body, in addition to be made from renewable resources [51].

No human clinical studies have yet examined rosin-based coatings on implants for corrosion protection as of the current date. The concept has been demonstrated only in laboratory and animal studies, where rosin films or blends applied to biodegradable metal implants slowed the corrosion rate and exhibited good biocompatibility. For example, rosin-coated polymers implanted subcutaneously in rats degraded over 2–3 months without causing significant inflammation, comparable to the biocompatibility of standard biodegradable polymers [52]. This finding is encouraging but purely preclinical. Clinical trials are still required to verify that rosin-based implant coatings can safely and effectively improve implant longevity in patients.

### 4.4. Remineralization-Promoting Tooth Varnish

Dental caries is one of the most common chronic childhood diseases caused by excessive demineralization of the teeth surfaces. The condition can be repaired by applying remineralization-promoting varnishes on the damaged surface. Gum rosin is a popular material used as a matrix of tooth varnish, carrying fluoride as a remineralization-promoting agent. Due to higher sensitivity towards fluoride found in children, some researches were performed to find safer remineralization promotors. It was found that gum rosin varnish containing peptide, bioactive glass, and egg shells performed similar mineralization promotion to fluoride-containing varnishes [53,54,55].

Rosin is already utilized in established dental treatments aimed at remineralization, although primarily as a carrier medium rather than the active remineralizing agent. Notably, common fluoride varnishes (e.g., 5% sodium fluoride Duraphat) contain a rosin-based resin to help the fluoride adhere to tooth enamel [56]. These rosin-containing varnishes have demonstrated clinical efficacy in promoting enamel remineralization and preventing caries as part of routine dental care. However, any novel remineralization strategies that rely on modified rosin compounds (beyond serving as a fluoride carrier) have yet to be validated in clinical trials.

### 4.5. Pharmaceutical Dosage Forms

The primary goal of drug delivery is to maximize therapeutical effect, minimize adverse effects and improve patients’ compliance by delivering the drugs directly to the site of action. The types of active pharmaceutical ingredients (API) have evolved from small molecules (less than 900 Daltons) to more complicated matters as peptides, proteins, antibodies, nucleic acids, and living cells. Each of these materials has specific characteristics, thus exhibit different challenges in formulating them into suitable drug delivery system [57,58]. The current trend towards utilization of sustainable materials in the development of biodegradable dosage forms has increased the interest in gum rosin as a potential excipients of drug delivery systems. This change is motivated by the growing necessity to reduce the reliance on petroleum-based synthetic polymers and harmful substances [59,60]. Some research examined the modification of abietic acid plentifully found in gum rosin [61]. Various reports described the use of gum rosin and its derivatives in drug delivery system, most were designed for small molecules drugs, ranging from relatively simple mechanisms to the more sophisticated ones. These works are explained in a dedicated section below.

The translation of rosin-based excipients into clinical use within pharmaceutical dosage forms remains limited. Numerous formulation studies highlight rosin’s utility in controlled-release tablets, microparticles, and transdermal systems, but these advances have largely not progressed beyond preclinical evaluation. In recent years (2020–2025), there have been no major clinical trial reports of novel rosin-containing drug delivery systems, indicating that their purported benefits (e.g., improved drug release profiles) are supported mainly by in vitro and in vivo data so far. Consequently, rosin’s role in approved pharmaceutical products is still relatively minor, and further clinical research is needed to establish its safety and efficacy in human therapy.

## 5. Drug Delivery System

Innovations in drug delivery systems (DDS) have continuously advanced to improve therapeutic efficacy and patient compliance. A study performed by Tewabe et al. (2021) provides a framework for understanding the development of drug delivery technology, started from Generation 1: Conventional dosage forms: capsule, tablet, emulsion, suspension; Generation 2: Modified action systems: enteric coating, repeat/prolong action; Generation 3: Controlled delivery systems: osmotically swelling and diffusion controlled systems; Generation 4: Targeted delivery systems: targeted, modulated, self-regulated delivery systems; and the advanced Generation 5: nanorobots, gene therapy, biologicals, long-term delivery systems [62]. Various materials have been used as excipients, and there is a growing trend in using natural resources in this application, including gum rosin. It can serve as a versatile film-forming material, providing film coatings enable sustained drug release. Gum rosin also plays a crucial role in the formation of microparticles with varied properties, from enhanced thermal stability to adjustable characteristics suitable for controlled-release drug administration and encapsulation. A study by Rosa-Ramirez (2023) showed that gum rosin esters proved to be effective, bio-based processability aids, offering enhanced flow properties and modest thermal and mechanical improvements with minimal downsides [63]. Its multifaceted properties, biocompatibility, cost-effectiveness, and ecofriendly nature further highlight its potential in the development of drug delivery systems. Various scientific papers reported the application of gum rosin and its derivatives in various drug dosage forms, includes plasters and ointments, matrix-forming in sustained-release tablet or medicated chewing gum (Generation 2), film-forming in transdermal film, coating agent in enteric-coated, sustained-released or microparticles dosage forms (Generation 3) and excipients for advanced drug delivery systems (Generation 4).

### 5.1. Taste-Masking Agent

Many orally administered drugs have unpleasant flavor, suggesting the need of taste-masking agents. Gum rosin is widely known as a nontoxic and hydrophobic substance; thus, it can serve as film coating materials for taste-masking purposes. In a study, gum rosin, in combination with ethyl cellulose and PEG 400 as a plasticizer, was utilized as a taste-masking film for ambroxol hydrochloride, an anti-mucolytic medicine. The study successfully produced smooth-surfaced microspheres with enhanced taste-masking characteristics and consistent diameters. This innovative approach proved that gum rosin is potential as coating excipient to improve the taste acceptability of pharmaceutical substances, offering a potential solution for the development of patient-centric, and enhancing patient adherence to medication [64,65].

### 5.2. Coating Materials for Controlled-Release Dosage Forms

**Figure 4:**
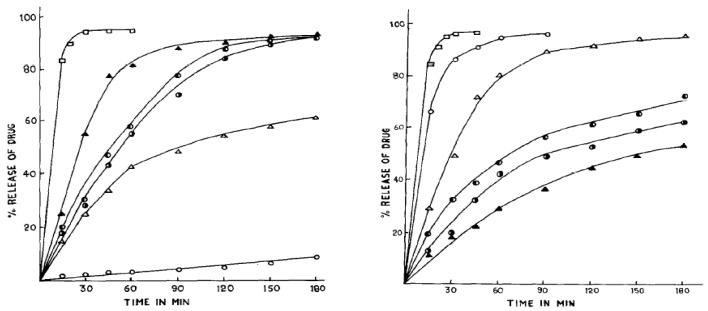
Study of coated drug dissolution in gastric fluid and intestinal fluid at 37 °C. Key: control (□); rosin-coated (○); RPE-coated (▲); RMEG-coated (◑); RMEP-coated (◐); abietic acid-coated (∆). Reproduced from ref (Pathak and Dorle, 1987) [69] with perm with permission. Copyright © 1987, Elsevier.

Chen et al. (2022) reported a durable, fluorine-free superhydrophobic coating made from sulfhydryl-modified rosin acid and silica nanoparticles (SiO_2_) [66]. Rosin’s natural phenanthrene ring structure enhances chemical stability and mechanical strength without the environmental risks posed by fluorinated materials. These attributes position rosin-based superhydrophobic coatings as promising candidates for advanced applications in healthcare, including anti-fouling medical devices and antimicrobial dressings, where low toxicity and biodegradability are necessary [66]. In similar study, Zaoui et al. (2020) found that rosin’s natural hydrophobic and renewable characteristics could be improved for sustainable applications, including the possibility of continuous processing and use in biomedical contexts such as tissue engineering [67]. Pathak et al. (1985) demonstrated that esterification of rosin with polyols such as glycerol, mannitol, or sorbitol produced effective film coatings that modulated the dissolution rate of aspirin tablets in simulated gastric and intestinal fluids [68]. In subsequent studies, the same group compared various coating excipients, namely unmodified rosin, rosin-pentaerythritol-ester (RPE), and rosin maleic acid adducts esterified with glycerol (RMEG) or pentaerythritol (RMEP). Their experiments revealed that both rosin and RPE provided exceptional moisture protection and unique drug release patterns (see Figure 4) with RPE emerging as the most satisfactory coating for controlled release due to its superior ability to retain drug release in the intestinal medium [69]. A recent study by Burakele (2023) showed that rosin films, particularly when combined with plasticizers, demonstrated suitability for coating captopril tablets, allowing for delayed drug release [70]. The coated tablets exhibited slow, controlled drug release for up to 8 h, ideal for chronotherapy targeting hypertensive crises, with stability studies confirming the formulations’ robustness over time [70]. Wang et al. (2024) also found rosin-apatite hybrids demonstrate high adsorption capacity for phenol and a good drug loading capacity for adriamycin through π–π stacking interactions with the rosin moieties [71].

### 5.3. Medicated Chewing Gum

Medicated chewing gum (MCG) has gained attention in drug delivery applications, as highlighted by a review from Thivya et al. (2021) [72]. This form of drug delivery has a broad range of applications, including drug delivery and nutraceuticals, with increasing demand, particularly for conditions related to oral hygiene and bad breath caused by smoking. MCGs offer non-invasiveness, easy administration, and faster metabolism through the liver or gut wall, making them popular for targeted drug delivery. MCGs, defined as a form of solid that can be chewed but not swallowed, contain bioactive or pharmaceutical compounds coated with a masticatory gum base. These compounds are released during the chewing process and absorbed in the oral mucosa, providing drug release for various oral diseases. Challenges in MCGs include factors affecting the release of active substances, such as membrane thickness, environmental conditions, chewing time, and solubility. Additionally, individual variability and formulation factors influence the efficiency of release. Despite these challenges, MCGs demonstrate efficacy in drug delivery, offering a fast onset time for systemic effects and reducing gastrointestinal and hepatic first-pass metabolism. Gum rosin played role as elastomer excipient in this type of dosage form [72].

Another study by Pandit and Joshi (2006) developed a chewing gum formulation designed for buccal delivery of diltiazem hydrochloride, aiming to bypass first-pass metabolism and achieve rapid onset of action [73]. They synthesized an ester derivative of rosin (a hydrophobic gum base) and incorporated the drug along with plasticizers (soybean oil or castor oil), beeswax, and sweeteners. In vitro and in vivo tests. Measuring drug release into saliva and monitoring urinary excretion showed that diltiazem hydrochloride was substantially absorbed via the buccal route within 15 min of chewing, with minimal residual drug in the gum [73]. The findings suggest that medicated chewing gum using rosin derivatives can be a novel, convenient system for delivering diltiazem hydrochloride quickly and effectively, potentially lowering the required dose and reducing hepatic first-pass effects.

### 5.4. Transdermal Drug Delivery System

Skin is the largest organ in human body; thus, it can serve as an alternative absorption site. Transdermal administration is comfortable for patients and avoids first-pass metabolism of the drugs. Despite its good film-forming ability, gum rosin is rarely used in transdermal drug delivery system, probably due to its dermal toxicity nature. However, this problem can be addressed by tailored modifications of the material. In a study by Satturwar et.al. (2005), polymerized gum rosin-derived abietic acid (PR) was used in the formulation of matrix-type transdermal drug delivery systems, with the goal of achieving controlled drug release. The challenges addressed included the need for flexible films with enhanced mechanical characteristics that could modulate drug release kinetics and transdermal penetration. To address these challenges, the researchers combined PR, polyvinylpyrrolidone (PVP), and dibutyl phthalate to make flexible film matrices containing a model drug diltiazem hydrochloride (DTH). PR tended to retain the drug while PVP released the drug more easily. The modulation of drug and PR-PVP composition allowed for the regulation of drug release kinetics and transdermal penetration from these films. This methodology presents a promising opportunity for the advancement of transdermal patches, offering the potential for assessing pharmacokinetics and pharmacodynamics in suitable animal models [9].

### 5.5. Micro- and Nanoparticles

The invention of medications in micro- and nanoparticle forms is revolutionary, because they are capable of high concentration of drug active agent uptake in their matrix and enables the long-acting medicines with release period up to six months. Long-acting strategy reduces the frequency of medication, thus improving patients’ comfort and compliance. The micro- and nano-sizes also enable, although not limited to, drug administration via injections, thus avoiding possibility of degradation when the drugs are administered orally [57,58]. In vitro drug release studies demonstrated that nanoparticles incorporating gum rosin provided a controlled and sustained release of fluvastatin sodium over 48 h. In vivo evaluations further supported these findings, as the rosin-containing formulations effectively reduced serum triglyceride and cholesterol levels in hyperlipidemic rat models [74]. Joshi and Sing (2020) developed a gelatin–rosin gum complex nanoparticles (GGR NPs) via a complex coacervation method followed by glutaraldehyde crosslinking, designed for colon-targeted delivery of 5-fluorouracil (5-FU) [15]. The optimized nanoparticles were confirmed have a mesoporous and amorphous structure that facilitates controlled drug encapsulation. In vitro release studies in simulated gastrointestinal fluids demonstrated sustained 5-FU release over 16 h, following first-order kinetics and a non-Fickian diffusion mechanism, while cytotoxicity assays showed enhanced efficacy of the drug-loaded nanoparticles.

Another study focusing on breast cancer therapy also applied the 5-fluorouracil (5FU) loaded PEG/rosin-pentaerythritol-ester (RPE). In vitro tests on breast cancer cell lines (MCF-7 and MDA-MB-231) revealed significantly higher cytotoxicity of 5FU-NPs and CAR-NPs at lower concentrations than free 5FU or CAR, while normal HUVEC cells remained largely unaffected. Mechanistic assays further demonstrated that ceramidase (ASAH1) and sphingosine-1-phosphate (S1P) expression decreased, alongside reduced Bcl-2, suggesting enhanced induction of apoptosis [75]. In studies focusing on metabolic disorders, Thymoquinone (TQ)-loaded gum rosin nanocapsules, when used in combination with other agents like glycyrrhizin, demonstrated significant antihyperglycemic effects in diabetic rat models by effectively reducing blood glucose levels, glycation of hemoglobin, and improving lipid profiles [76]. Singh and Malviya (2018) presented a novel strategy to enhance the delayed, colon-specific release of 5-aminosalicylic acid (5-ASA) by creating hybrid nanoparticles composed of carboxymethyl cellulose (CMC) and gum rosin [77]. The result showed the potential of the CMC–gum rosin hybrid nanoparticles to delay drug release until the lower GI tract, thereby enhancing local drug bioavailability.

### 5.6. Targeted Drug Delivery Systems

Targeted drug delivery is one of the most sophisticated strategy in the drug delivery system [62]. It is one of the realizations of the “magic bullet” concept introduced by Paul Ehrlich, a winner of Nobel Prize for Physiology or Medicine in 1908. In this concept, drugs can be delivered exactly to the targeted area in the body without affecting healthy cells, tissues, or organs. Hence, the amount of the required medicine can be lowered, and the adverse effect to healthy parts can be minimized [62,78]. The ability of gum rosin and its derivates as film-forming and matrix-forming excipients allowed this material to produce nano- and microparticles, not only for oral administration, but also for parenteral-targeted drug delivery system. Various active pharmaceutical ingredients (API) have been studied, and a series of studies have explored the versatility of rosin and its derivatives in the field of targeted drug delivery.

#### 5.6.1. Targeted Delivery for Anticancer Drugs

**Figure 5:**
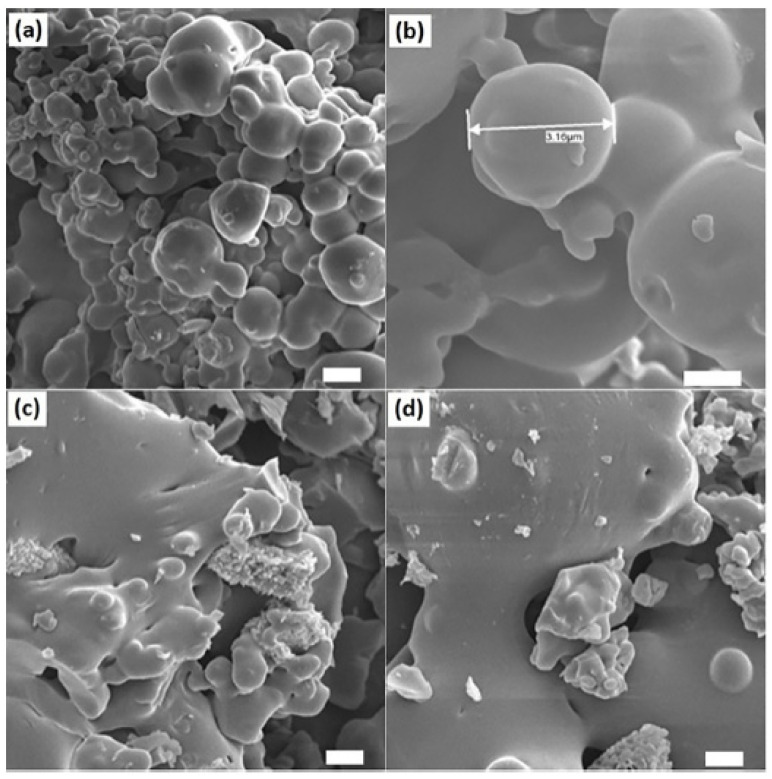
SEM micrographs of drug-loaded GR/PEO microspheres (**a**,**c**) and placebo GR/PEO microspheres (**b**,**d**) at two different resolutions (Scale bar = 1 µm). Reproduced with permission of ref (Madhavi et al., 2023) [79]. Copyright © 2023, Elsevier.

Gum rosin was studied for its potential as carrier for anti-cancer/chemotherapy drugs. Joshi and Singh (2020) incorporated 5-Fluorouracil (5-FU) into gelatine-rosin gum nanoparticles (GGR NPs) and found that 5-FU GGR NPs exhibited first-order kinetics drug release profile and a combination of diffusion and erosion mechanisms [15]. The network configuration of the nanoparticles facilitated a sustained release of the drug, and cytotoxicity evaluations on A549 (lung cancer) cells demonstrated higher toxicity of 5-FU-laden nanoparticles compared to those without drug loading. These findings suggest that the modification of gum rosin in the form of GGR NPs offers a viable and secure approach for delivering chemotherapeutic drugs, with the potential to improve effective effects [15]. Madhavi et al. (2023) introduced a novel controlled-release mechanism for the anticancer agent 6-Thioguanine (6-TG). In this study, 6-Thioguanine (6-TG) was encapsulated in biodegradable microsphere of gum rosin (GR) and poly (ethylene oxide) (PEO) using double emulsion solvent evaporation technique [79]. Microspheres with and without the active agent were successfully made, as shown in Figure 5. The microspheres demonstrated encapsulation efficiencies ranging from 64.32% to 72.42% and the in vitro release profiles exhibited diverse patterns, suggesting the potential for tailoring drug delivery profiles for specific requirements [79].

#### 5.6.2. Targeted Delivery of 5-ASA, an Anti-Inflammatory Drug

Singh et al. (2018) [77] utilized gum rosin to deliver and control release mechanism of 5-aminosalicylic acid (5-ASA), an anti-inflammatory medication commonly used for ulcerative colitis treatment. 5-ASA was encapsulated in synthesized hybrid nanoparticles known as carboxymethyl cellulose-rosin gum nanoparticles (CRNP3) [71]. These nanoparticles exhibited a controlled release of 72% of the drug over 12 h when exposed to simulated intestinal fluid, contrasting with the complete release of 100% observed between 5 to 8 h from the original materials.

**Figure 6:**
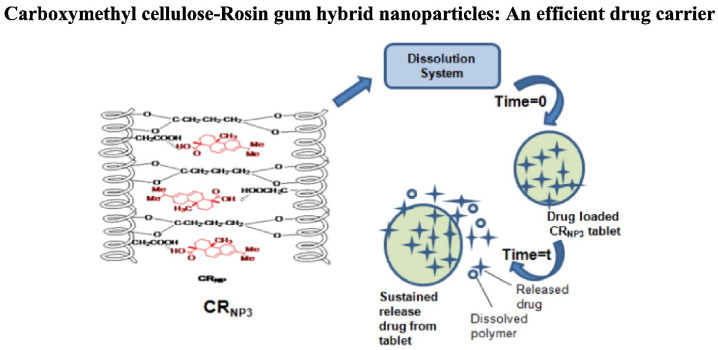
Targeted drug delivery of 5-ASA, an anti-inflammatory drug. Reproduced from ref (V. Singh et al., 2018) [77] with permission. Copyright © 2018, Elsevier.

Controlled and delayed release mechanisms were identified as advantageous for enhancing the bioavailability of 5-ASA, particularly in the colon. The modification of gum rosin in the form of CRNP3 nanoparticles demonstrated zero-order kinetics and a non-Fickian diffusion mechanism, indicating the potential effectiveness of these hybrid nanoparticles as a targeted drug administration method in the gastrointestinal tract. The concept of this work is depicted in Figure 6 [77].

#### 5.6.3. Targeted Delivery of Periodontitis Medication

**Figure 7:**
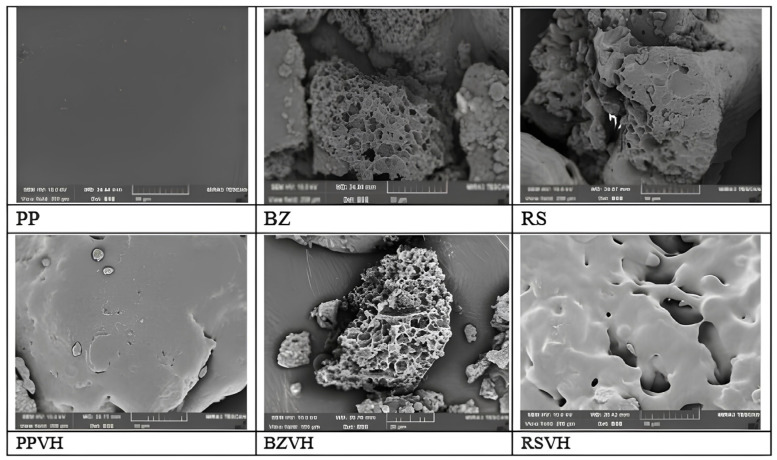
SEM micrographs of dry remnants of drug free and VH-loaded in situ forming gel systems (1000×). Reproduced from ref (Khaing et al., 2021) [80] with permission. Copyright © 2021, MDPI.

Besides its film-forming ability, gel-forming properties of rosin has been also investigated for its application to form in situ gel for periodontal disease. Khaing et al. (2021) explored the use of rosin (RS) along with other natural resins including benzoin (BZ) and propolis (PP), in the development of in situ forming gels (ISGs) as a delivery method for vancomycin hydrochloride (VH) [80]. A total of 35% *w*/*v* of the resins were dissolved in DMSO and then subjected to solvent exchange procedure with water to form the gels. The study found that the rosin-based ISGs exhibited a pseudo-plastic flow characteristic, contributing to the injectability and mechanical properties. However, in comparison to benzoin and propolis-based ISGs, rosin-based ISGs slower release of VH. SEM images of dried gels based on RS and BZ indicated the presence of homogeneous porous system, while pores did not appear on PP-based gels (Figure 7). The images of dried VH-loaded gels after drug release showed that the BZ-based gel contained more pores than the RS-based gel, indicating that more VH left the BZ-based ISG than the other. VH was deposited on the surface of PP-based ISG, enabling its relatively easy release [80].

**Figure 8:**
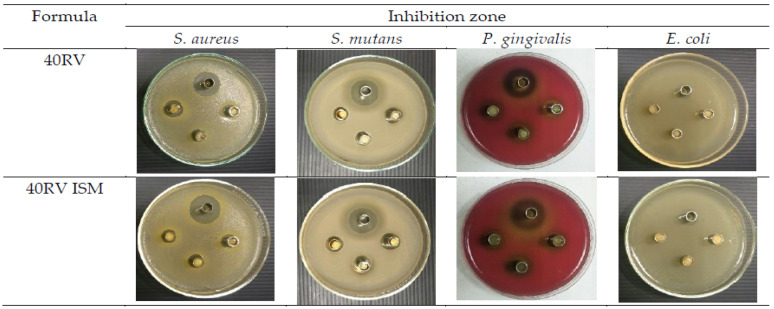
Photographs of the inhibition zone of 40RV and 40RV ISM formulations against *S. aureus*, *S. mutans*, *P. gingivalis*, and *E. coli* (n = 3) and the control group (VD) at upper side cup. Reproduced with permission of ref (Chuenbarn et al., 2022) [81]. Copyright © 2022, MDPI.

The study to utilize rosin as an ISG was continued by the same group, as reported by Chuenbarn et al. in 2022 [81]. Rosin was chosen because it prevented initial VH release, which is important to prevent side effects and antibiotic resistance [81]. This study successfully showed the effectiveness of rosin-based ISG and in situ forming microparticles (ISM) as localized delivery vehicles for VH. ISG was made using the same procedure as the previous study [80] with varied rosin concentration. ISM was produced from a mixture of rosin solution with a solution of glyceryl monostearate in olive oil as an external oil phase. The study found that both ISG and ISM exhibited a pH range from 5.02 to 6.48, ease of injectability, and satisfactory stability, with ISM showing enhanced injection performance due to the lubricating properties of the external oil phase. The modification of gum rosin in these systems resulted in formulations that demonstrated flexibility throughout the phase change process, allowing them to conform to the contours of a patient’s gum cavity. Moreover, the concentration of rosin influenced the size of microparticles, and formulations with higher rosin concentrations exhibited higher ability to delay the drug release. Figure 8 shows the bacteria-inhibiting test of ISG with 40% rosin (40RV) and ISM with an equivalent rosin concentration (40RV ISM), compared to a saline solution (yielding in large inhibition areas). The small inhibition areas made by the ISM and ISG demonstrated the delayed drug release, which were effective against *S. mutans* and *P. gingivalis* [81].

**Figure 9:**
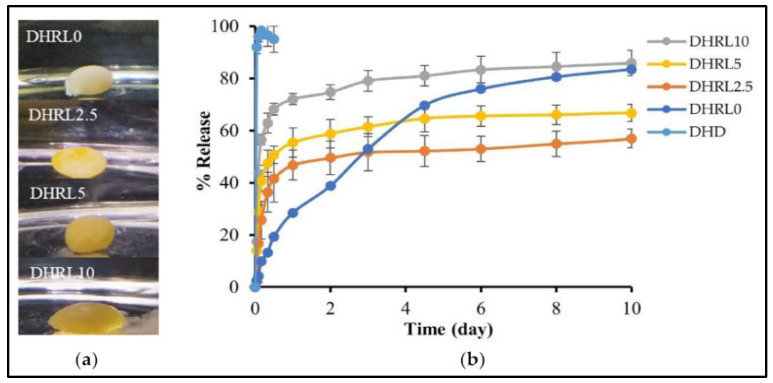
DH-loaded rosin in situ forming a gel with various concentrations of LO: (**a**) Gel formation in the release medium after one hour; (**b**) DH release behavior on oil/rosin in situ forming gel over ten days (n = 3). Reproduced from ref (Khaing et al., 2022) [82] with permission. Copyright © 2022, MDPI.

Rosin-based in situ forming gel was also used in delivering doxycycline hyclate (HC) and lime peel oil as a prospective injectable therapy for localized treatment of periodontitis. 55% *w*/*w* of rosin was dissolved in DMSO and N-methyl pyrrolidone (NMP) solvents, along with doxycycline hyclate and varied amount of lime peel oil. As shown in Figure 9, the inclusion of LO was seen to decelerate the process of gel formation, marginally elevate viscosity and injectability, diminish gel hardness, and augment adhesion. The drug release exhibited a non-Fickian diffusion mechanism and demonstrated greater efficacy in the formulation with LO addition throughout a 10-day timeframe. Significantly, the incorporation of a 10% LO concentration demonstrated enhanced antibacterial properties against *Porphyromonas gingivalis* and *Staphylococcus aureus* in the formulation, suggesting the potential utility of LO in the efficacious management of periodontitis [82].

Although gum rosin and its derivatives exhibit biological activities in vitro, their main function in pharmaceutical products is to serve as versatile, tunable excipients. Gum rosin’s value in formulation science lies in the ability to be modified or blended to achieve desired drug delivery characteristics, whereas its development as a standalone active pharmaceutical ingredient (API) remains limited. In practice, gum rosin continues to be utilized chiefly as a formulation aid, enhancing properties like release kinetics and palatability, rather than as a direct therapeutic agent in clinical use.

## 6. Challenges of Gum Rosin as Pharmaceutical Excipient for Frug Delivery System

### 6.1. Allergenic Activity and Occupational Exposure

The allergenic potential of gum rosin is primarily attributed to the presence of oxidized resin acids, especially those of the abietadiene-type, that form when rosin is exposed to air. Karlberg et al. (1988) identified 15-Hydroperoxyabietic acid, an oxidation product, as a prominent allergen in unaltered gum rosin [83]. Several subsequent studies have successfully isolated and identified various oxidized resin acids as contact allergens [84,85,86,87,88,89]. Although chemical modifications such as hydrogenation and esterification with polyalcohols can reduce the sensitizing potential of rosin, concerns regarding the allergenic effects of unmodified rosin persist in many products even after such modifications [85,90,91,92,93]. Allergic contact dermatitis (ACD) associated with rosin, commonly known as colophonium in the International Nomenclature of Cosmetic Ingredients (INCI), is a well-documented adverse reaction. In clinical settings, patch testing with a 20% concentration of gum rosin in petrolatum is routinely used to identify individuals sensitive to rosin [92]. Epidemiological studies have reported a prevalence of rosin hypersensitivity ranging from 0.45% to 2.0% in various European populations, with data from Germany indicating rates between 0.6% and 1.4% over a 10-year period. Moreover, several studies from Denmark, Sweden, and other European countries have noted a higher incidence of rosin allergy in teenagers and older individuals, likely due to prolonged or repeated exposure [94,95,96,97,98].

Occupational exposure further exacerbates the risk of rosin-induced allergic reactions. Workers handling rosin-containing products (such as adhesives, leg ulcer treatments, epilating waxes, cosmetics, and shoe lining), are particularly vulnerable to developing ACD [99,100,101,102,103,104]. Additionally, musicians (especially string and percussion instrument players), soldering workers in the electronics industry, textile workers, and machinists exposed to rosin in metalworking fluids face an increased risk of contact dermatitis. The potential for exposure is also elevated in industries dealing with paper products, diapers, and sanitary pads, as well as in certain wooden items like toilet seats [105,106,107]. Furthermore, inhalation of rosin fumes has been linked to occupational asthma; for instance, Elms et al. (2005) [108] reported that approximately 5% of newly diagnosed cases of occupational asthma in the United Kingdom in 1994 were related to exposure to rosin-containing solder vapors. This respiratory reaction is thought to be mediated by IgE antibodies, which can induce airway inflammation and bronchoconstriction [108].

Several strategies have been employed to address rosin’s allergenicity. One effective approach is chemical modification of rosin to remove or alter the allergenic components. Hydrogenation of gum rosin is a prime example. By hydrogenating rosin, the double bonds in abietic-type acids (which are prone to oxidation) are saturated, yielding hydrogenated rosin (often called “staybelite” resin when fully hydrogenated). Hydrogenated rosin contains mostly dihydroabietic and tetrahydroabietic acids, which are far less likely to oxidize into allergenic species [109]. Previous study has shown that hydrogenated rosin has reduced sensitizing capacity compared to unmodified rosin [110]. In practical terms, adhesives formulated with hydrogenated rosin or certain rosin esters cause fewer allergic reactions, and these modified rosins are already used in products like hypoallergenic bandages and medical tapes. Another strategy is purification. Removing trace metal impurities (that might catalyze oxidation) and eliminating known irritant fractions from rosin can lower its allergenicity. For example, refined rosin where oxidation products have been distilled out has a cleaner profile and is less likely to cause allergy [111]. Lastly, use-level control is a simpler approach. By using the minimum effective amount of rosin in a formulation and encapsulating it within polymer matrices (so that free rosin is not directly in contact with tissues), the exposure of patients to allergenic components can be minimized [1].

### 6.2. Brittleness and Mechanical Weakness

Gum rosin is a hard and brittle resin at room temperature. While this property is useful for creating stiff films or coatings, it poses a challenge when flexibility and mechanical strength are required. For example, in tablet coatings that must withstand handling, or in transdermal patches that must bend with skin. A pure rosin film tends to crack or shatter under stress because of rosin’s glassy nature (glass transition typically near ambient temperature). This brittleness is mainly due to rosin’s low molecular weight (around 302.5 g/mol for abietic acid) and lack of flexible polymeric chains (National Center for Biotechnology Information, 2025). If a rosin-based matrix cracks, it can compromise the drug release profile (causing dose dumping) or, in the case of a patch, cause the patch to lose adhesion.

To overcome brittleness, researchers have turned to plasticizers and polymer blending. The addition of plasticizers is a common solution. By incorporating a plasticizer such as dibutyl phthalate, triethyl citrate, or other compatible softening agents, the rosin matrix becomes more flexible and less prone to cracking. In the earlier mentioned transdermal patch study, adding 30% *w*/*w* of dibutyl phthalate to polymerized rosin was crucial in obtaining a flexible film [9]. Plasticizers work by inserting themselves between rosin molecules, reducing intermolecular forces and effectively lowering the glass transition temperature of the rosin film. Another, often complementary, strategy is blending rosin with other polymers that provide elasticity or toughness. For instance, rosin has been blended with elastomeric polymers like poly (ethylene-co-vinyl acetate) (EVA) or with other natural polymers to improve film properties. In one formulation, a blend of gum rosin with poly (ethylene oxide) (PEO) was used to create microspheres. PEO, being flexible and hydrophilic, helped to counteract rosin’s brittleness while also modulating drug release [79]. Similarly, blending rosin with polyvinylpyrrolidone or small amounts of cellulose derivatives can yield a matrix that is both strong and pliable. Yet another approach is using derivatized rosin with inherently better mechanical properties. As noted, polymerized rosin already provides improved tensile strength relative to unmodified rosin because the polymerization process creates larger molecular network [9]. Rosin can also be turned into acrylate or methacrylate monomers (by reacting rosin acids with acrylic/methacrylic groups) and then polymerized into higher molecular weight polymers that are less brittle [112]. These rosin-based polymers behave more like traditional plastics or resins, offering greater durability.

### 6.3. Hydrophobicity and Drug Release Modulation

The strong hydrophobic nature of gum rosin has advantages as well as disadvantages. On the one hand, it is the basis for rosin’s utility in protecting against moisture and in sustaining drug release. On the other hand, excessive hydrophobicity can be a drawback when a formulation requires a degree of water interaction (for example, to release a drug or to biodegrade over time). If a rosin-based matrix is too impermeable to water, a drug embedded in it may release too slowly or not at all (leading to incomplete release). Moreover, highly hydrophobic materials may persist in the body for long periods, which is a concern if they are not intended to be permanent implants. Thus, a key challenge is tuning the hydrophobicity of rosin-based excipients to an optimal level for the intended application.

The main strategy to deal with rosin’s hydrophobicity is by introducing hydrophilic components or functional groups into the rosin matrix. One straightforward method is polymer blending with hydrophilic polymers. By mixing rosin with a water-soluble or water-swellable polymer (e.g., PEO, PVP, polyethylene glycol, or certain grades of hydroxypropyl methylcellulose), the resultant matrix can absorb some water and create channels for drug release. The blend used in rosin–PEO microspheres, for instance, enabled a more controlled and complete drug release compared to pure rosin microspheres [79]. The hydrophilic polymer portion can dissolve or swell upon contact with bodily fluids, increasing the porosity of the rosin matrix and thereby facilitating drug diffusion.

### 6.4. Other Challenging Characteristics

Besides its potentially allergic-inducing nature, gum rosin also exhibits several challenging characteristics. Its melting point of 70–85 °C and its hydrophobic nature provides suitable characteristics as matrix and coating materials for various controlled-release dosage forms includes tablets, microcapsules, nanoparticles and films. However, unmodified rosin film was found to be too hydrophobic, too brittle, and containing pores that allow for relatively high water-vapor transmission rate when applied as drugs coating [68]. Gum rosin is able to retain the drug release up to more than 24 h. This may lead to over-retained drug release from the dosage forms and cause insufficient drug concentration in the site of action/site of absorption. Therefore, gum rosin was never applied as a single excipient, especially for oral-administered dosage forms. Furthermore, it is important to modify its structure to improve the functional characteristics of gum rosin.

## 7. Strategies to Optimize the Application of Gum Rosin

In order to expand the application of gum rosin, its functional characteristics should be improved. There are several strategies can be employed, including performing physical and chemical modification on gum rosin. Combining gum rosin with other polymers such as ethyl cellulose [64] and polyvinylpyrrolidone (PVP) [9] have been proved to be effective to improve functional characteristics the obtained film. Furthermore, combinations of gum rosin with gelatine [15] produced nanoparticles with controlled-release drug profile, while its combination with carboxymethyl cellulose successfully produced colon-targeting dosage forms which exhibit zero-order kinetics and a non-Fickian diffusion mechanism [77]. Addition of plasticizer such as PEG 400 or dibutyl phthalate successfully further improved the flexibility of the film matrices. Chemical modification of gum rosin further improved its characteristics. Modification of gum rosin into rosin-pentaerythritol-ester (RPE) resulted in coating film with exceptional moisture protection properties and satisfactory controlled-release profile in the simulated intestinal fluid [69]. These studies showed that physical and chemical modification on gum rosin may result in materials with better characteristics, thus open new opportunities to use these modified substances for broader applications in pharmaceutical fields.

## 8. Conclusions

In conclusion, rosin, a natural resin extracted from pine trees, has proven to be a remarkably versatile and valuable resource across various industries. Its applications in textiles have led to the development of antimicrobial cotton textiles, green biopolymer packaging nanocomposites, and protective coatings for wood, enhancing durability and functionality. Within the pharmaceutical field, rosin’s significance is highlighted by its role in controlled drug delivery, enabling precise regulation of drug release, as well as in targeted drug delivery systems with tailored release profiles. Furthermore, rosin-based nanogels have demonstrated potential in the development of effective antimicrobial drugs. Rosin’s natural origin, biocompatibility, and diverse range of properties position it as a key asset with multifaceted utility, contributing to advancements in both textile and pharmaceutical industries. Physical and chemical modification on gum rosin might improve its characteristics to be suitable for various dosage forms. Its continued exploration and innovative applications hold promise for further advancements in these fields and beyond.

## Figures and Tables

**Table 1:** Summary of general applications of gum rosin.

Application Area	Function/Role	Key Benefits/Properties	References
Adhesives and Tackifiers	Acts as a tackifier in hot-melt and pressure-sensitive adhesives; also used as a grip enhancer (e.g., in sports and dance floors).	Improves initial stickiness by enhancing wetting and bonding; cost effective (accounts for 30–50% of formulation); enhances mechanical performance and adhesion.	[21,22]
Printing Inks	Binder/Film-Former in Offset, Heatset, and Package Printing.	Enhances pigment dispersion, adhesion, water resistance, gloss, quick-drying properties, and emulsification stability.	[23]
Paper and Packaging	Enhances wet strength in paper and is used in biodegradable packaging films and nanocomposites.	Increases paper hydrophobicity; packaging films exhibit improved tensile, viscoelastic, and antimicrobial properties along with enhanced barrier performance.	[24,25,26,27,28]
Rubber and Elastomers	Functions as an extender in synthetic rubbers and as a crosslinking agent in silicone rubber.	Improves processability, tensile strength, filler dispersion, and overall quality of vulcanizates; enhances thermal and mechanical properties in silicone rubbers.	[29]
Coatings	Utilized as a film-forming component in protective wood coatings.	Enhances adhesion and hardness; provides resistance to moisture and fungal decay through an effective barrier formation.	[30]
Food and Beverage	As a food additive primarily used as a stabilizer and emulsifier to maintain uniformity and prevent separation of ingredients.	Enhances the stability and consistency of emulsions in flavored drinks, improves the shelf life and appearance of food products, and complies with updated safety standards.	[31]
Textile Coatings	Applied as a protective coating on fabrics, such as personal protective clothing against pesticides.	Provides a hydrophobic barrier while maintaining acceptable breathability and comfort; performance comparable to commercial-grade PPC.	[32,33]
Industrial Applications	Enhances adhesion, flexibility, durability, and resistance properties across various industrial applications, such as adhesives, paints, inks, epoxy resins, plastics, paper sizing, surfactants, tires, insulation.	Improves adhesion, water resistance, printability, thermal stability, impact resistance, and chemical resistance.	[34]
Insecticidal Application	Used as an effective insecticide.	Have strong insecticidal efficacy against oriental armyworms, minimal toxicity toward aquatic organisms, and an ecofriendly, sustainable design.	[35]

## Data Availability

No new data were created or analyzed in this study. Data sharing is not applicable to this article.
